# Novel Human Artificial Intelligence Hybrid Framework Pinpoints Thyroid Nodule Malignancy and Identifies Overlooked Second-Order Ultrasonographic Features

**DOI:** 10.3390/cancers14184440

**Published:** 2022-09-13

**Authors:** Xiaohong Jia, Zehao Ma, Dexing Kong, Yamin Li, Hairong Hu, Ling Guan, Jiping Yan, Ruifang Zhang, Ying Gu, Xia Chen, Liying Shi, Xiaomao Luo, Qiaoying Li, Baoyan Bai, Xinhua Ye, Hong Zhai, Hua Zhang, Yijie Dong, Lei Xu, Jianqiao Zhou, CAAU

**Affiliations:** 1 Department of Ultrasound, Ruijin Hospital, Shanghai Jiaotong University School of Medicine, Shanghai 200025, China; jxh12022@rjh.com.cn (X.J.); dyj11584@rjh.com.cn (Y.D.); 2 School of Mathematical Sciences, Zhejiang University, Hangzhou 310013, China; 21835035@zju.edu.cn (Z.M.); dxkong@zju.edu.cn (D.K.); 3 Zhejiang Qiushi Institute for Mathematical Medicine, Hangzhou 311121, China; 4 College of Mathematical Medicine, Zhejiang Normal University, Jinhua 321004, China; 5 Department of Ultrasound, Puyang People’s Hospital, Puyang 457005, China; pysrmyybgs@163.com; 6 Demetics Medical Technology, Hangzhou 310012, China; huhairong@demetics-medical.com; 7 Department of Ultrasound, Gansu Provincial Cancer Hospital, Lanzhou 730050, China; guanling1966@sina.com; 8 Department of Ultrasound, Shanxi Provincial People’s Hospital, Taiyuan 030012, China; 13593157054@163.com; 9 Department of Ultrasound, The First Affiliated Hospital, Zhengzhou University, Zhengzhou 450052, China; zhangruifang999@hotmail.com; 10 Department of Ultrasound, Affiliated Hospital of Guizhou Medical University, Guiyang 550001, China; gggu-ying@163.com (Y.G.); chenxia_gy@sina.com (X.C.); 2991238@163.com (L.S.); 11 Department of Ultrasound, The Third Affiliated Hospital of Kunming Medical University, Yunnan Cancer Hospital, Kunming 650031, China; blueskyluoxiaomao@163.com; 12 Department of Ultrasound Diagnostics, Tangdu Hospital, Fourth Military Medical University, Xi’an 710038, China; qiaoyingli@hotmail.com; 13 Department of Ultrasound, Affiliated Hospital of Yan’an University, School of Medicine, Yan’an University, Yan’an 716000, China; 13891103032@163.com; 14 Department of Ultrasound, The First Affiliated Hospital of Nanjing Medical University, Nanjing 210029, China; yexh-0125@163.com; 15 Department of Ultrasound, Traditional Chinese Medical Hospital of Xinjiang, Urumqi 830000, China; shuanghaizhai@163.com; 16 Department of Ultrasound, Anyang Tumor Hospital, The Fourth Affiliated Hospital of Henan University of Science and Technology, Anyang 455000, China; anyangzhanghua@163.com

**Keywords:** thyroid nodule, malignancy risk stratification, feature selection, second-order feature interaction, interpretable deep learning, radiology

## Abstract

**Simple Summary:**

Deep learning-based computer-aided diagnosis has gained momentum in the radiology field thanks to the technological advances of convolutional neural networks (CNN). However, how to utilize the black-box predictions of these CNN models to the clinical routine still relies on radiologists’ personal judgements. In addition, existing CNN models only improve radiologists’ diagnosis when they outperform the radiologists, thereby limiting their added values for possible efficiency enhancement and improving mostly the diagnostic performances of junior radiologists.

**Abstract:**

We present a Human Artificial Intelligence Hybrid (HAIbrid) integrating framework that reweights Thyroid Imaging Reporting and Data System (TIRADS) features and the malignancy score predicted by a convolutional neural network (CNN) for nodule malignancy stratification and diagnosis. We defined extra ultrasonographical features from color Doppler images to explore malignancy-relevant features. We proposed Gated Attentional Factorization Machine (GAFM) to identify second-order interacting features trained via a 10 fold distribution-balanced stratified cross-validation scheme on ultrasound images of 3002 nodules all finally characterized by postoperative pathology (1270 malignant ones), retrospectively collected from 131 hospitals. Our GAFM-HAIbrid model demonstrated significant improvements in Area Under the Curve (AUC) value (*p*-value < 10^−5^), reaching about 0.92 over the standalone CNN (~0.87) and senior radiologists (~0.86), and identified a second-order vascularity localization and morphological pattern which was overlooked if only first-order features were considered. We validated the advantages of the integration framework on an already-trained commercial CNN system and our findings using an extra set of ultrasound images of 500 nodules. Our HAIbrid framework allows natural integration to clinical workflow for thyroid nodule malignancy risk stratification and diagnosis, and the proposed GAFM-HAIbrid model may help identify novel diagnosis-relevant second-order features beyond ultrasonography.

## 1. Introduction

Ultrasound is the most widely used medical imaging tool to evaluate thyroid nodules, whose prevalence is found to be 30% to 68% [1,2], and the malignancy risks are evaluated based on the ultrasound nodular features according to Thyroid Imaging Reporting and Data System (TIRADS). While having many shared risk attributes, there exist TIRADS standards like ACR-TIRADS [3] proposed by American College of Radiology, Kwak-TIRADS [4] by Korean researchers, Eu-TIARDS [5] from the European Thyroid Association, K-TIRADS [6] from the Korean Society of Thyroid Radiology and the Korean Society of Radiology, as well as most recent C-TIRADS [7] proposed by the Chinese Medical Association and the Chinese Artificial Intelligence Alliance for Thyroid and Breast Ultrasound (CAAU). Despite being more standardized, no matter what TIRADS is taken for thyroid nodule screening, the lesion interpretations by radiologists are still subjective.

With the development of deep learning Convolutional Neural Networks (CNNs), radiologists start to use computer aided diagnosis (CADx) systems as a second opinion for thyroid nodule evaluation [8,9,10]. It consists of many layers of computations, taking the advantage of convolution computations over the images using kernels or filters of different sizes to extract features relevant to a classification task [11]. The outputs of each layer serves as the inputs for the next. The introduction of nonlinear activation functions in CNN models enables complex nonlinear mapping between their inputs and outputs. After many layers of computations, the activated spatial patterns of the perceptive field or the feature maps become incomprehensible to humans. Consequently, it is up to individual radiologists to make their final decisions. An immediate challenge facing the radiologists is how to objectively integrate such CADx systems into their clinical workflow such that the usage of these tools also becomes standardized. No less important is whether this integration can be made understandable or at least interpretable to humans. Otherwise, creating yet another black box on top of one does not address the concerns of radiologists as we would always prefer a conceptually meaningful explanation to an important decision. It is desirable to standardize the adoption of CADx systems in clinical practices by constructing a set of meaningful rules of criteria about how to use them in an objective way.

For medical image diagnosis, generating class activation heatmaps [12,13,14,15] localizes the deep representation of class-discriminating image regions of diseased tissues [9,16,17]. However, these techniques merely highlight the regions of importance for the class prediction with false color visualization (for instance, using typically red and blue respectively for positive and negative contribution), offering no further insight into what exact features from those outlined regions contribute to the model’s predictions. An overlooked possibility is to take the diagnosis results of a trained CNN as an input feature, aggregate it to human-extracted ones for subsequent feature selection and weight optimization to refine the final prediction. In essence, the malignancy score outputted by a CNN is assigned with a weight representing its contribution to the final decision. Though how the CNN as a modular building block of the complete decision-making system, which we refer as Human Artificial Intelligence hybrid (HAIbrid) framework, comes about its decision is still opaque, that is no longer the case with regards to its contribution. Consequently, it enables us to generate a new set of TIRADS criteria that sum up contributions of both features noted by radiologists and a CNN model for malignancy risk stratification and diagnosis. 

In this work, we chose Kwak-TIRADS as the basis for thyroid nodule evaluation as it has been widely adopted in China. In addition, we included extra vasculature-related ultrasound features [18] that describe both the localizations in relation to a nodule (i.e., being perinodular, close to the center, etc.) and their morphologies (being twisted or not twisted) to explore possibilities for establishing novel clinically-relevant TIRADS criteria. The complete set of candidate features is referred as TIRADS^+^ and listed in Appendix A Table A1. The interplay between cancerous tumors and vascularization is generally acknowledged in cancer research [19,20], and vascularity features observed in color Doppler images are an indispensable component in the T [21]. Some other malignancy-relevant features in ACR BIRADS, for instance posterior echoic features, heterogeneity of echo patterns, etc., are also included in the candidate feature set. In addition, the presence of hypoechoic or anechoic rim referred to as halo surrounding nodule introduced in C-TIRADS [7] and its thickness were also considered. 

Hypothetically, co-occurrence of features or second-order feature interactions can constituent a unique malignancy-relevant feature. Just as an individual’s inherent inter-dependent preferences over items of certain categories can be modeled by second or higher-order feature interactions using Factorization Machine (FM) [22] for improving the prediction of online users’ click-through rate for advertising [23], there presumably can also exist interdependence between certain ultrasound features of malignant thyroid nodules. However, to our humble knowledge, this has never been explored in the context of the CADx field. Additionally, as different feature interactions may have unequal impacts on predictions, on top of an existing attention-FM variant (AFM) [24] that weighs the importance of each feature interaction, we introduce an additional gate mechanism that removes the interference of minimally relevant feature interactions, termed GAFM, to explore the usefulness of second-order interacting features in ultrasound-based thyroid nodule diagnosis. 

As a proof of concept, we took a ResNet101 model [25] pretrained on ImageNet as a basis CNN and used the Area Under the Curve (AUC) of receiver operating characteristics as the main evaluation metric to compare the diagnosis results by standalone radiologists, CNN-based CADx, and by HAIbrid methods using feature selection modules of logistic regression (LR), conventional AFM model and our modified GAFM model. We compared two CNN systems integrated through our proposed GAFM-HAIbrid model, including an already-trained commercial system, to validate the usefulness of the model on an extra ultrasound image dataset of 500 nodules.

## 2. Materials and Methods

### 2.1. Clinical Samples

This study was based on the authorized clinical dataset with continuous data contribution from the consortium of CAAU [7] with 131 alliance hospitals led by Ruijin Hospital Affiliated to School of Medicine, Shanghai JiaoTong University. All registered data were from patients who had taken preoperative ultrasound examinations and had definitive postoperative pathological examination outcomes. The pathological results were taken as the gold standard for diagnosis evaluation by radiologists, CNN and the integrated approaches.

For each nodule, ultrasound scans of standard longitudinal and transverse planes were saved, including B-mode and color Doppler images. A retrospective cohort of 3002 thyroid nodules from 3002 patients continuously registered at CAAU alliance hospitals collected between January 2017 and August 2019 following the same protocol as in the previous publication was used for training and cross-validating our proposed model. Of these, 1732 nodules were determined to be benign, and 1270 nodules were malignant according to histopathological diagnosis. These samples were randomly partitioned into 10 folds for cross-validation experiments for which each fold was alternatingly taken as the validation set while the rest were taken as the training set. It is also important to note that dataset shift, which introduces substantial set to set variations, is a known deteriorating factor for accurate performance estimation [26]. To eliminate this effect, we adopted the distribution-balanced stratified cross-validation [27] algorithm to balance sample distributions in the feature space when partitioning a dataset according to radiologists’ assigned features. 

Data for an additional 500 nodules collected between February and April of 2021 were used to verify results obtained on the former cohort. Half of these nodules were benign and the other half were malignant, confirmed by pathological examinations. 

### 2.2. Candidate Feature Set Construction

**Figure 1:**
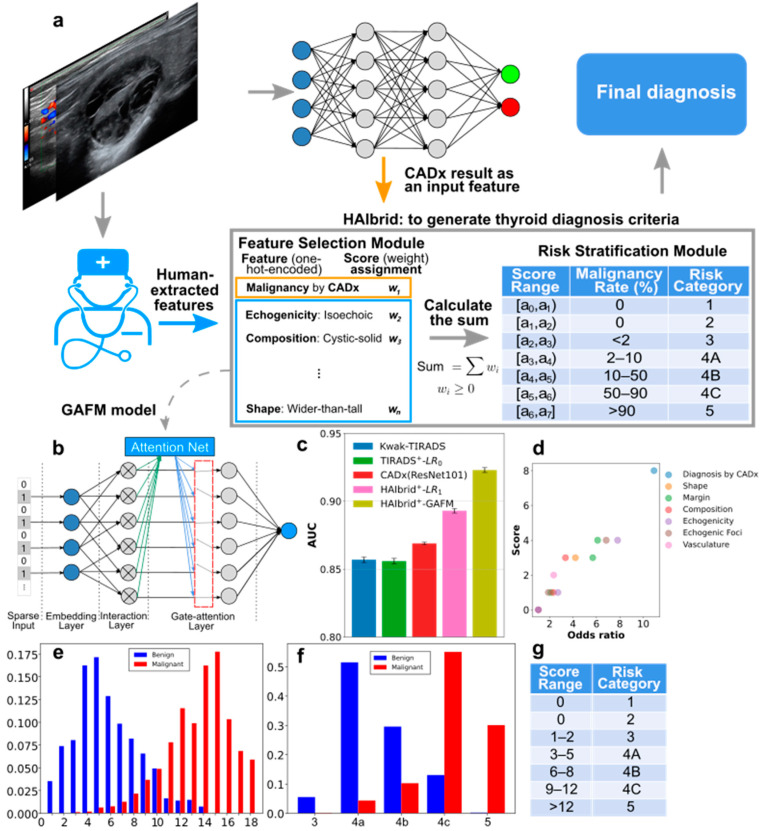
Our proposed HAIbrid method to establish CNN-boosted human-understandable thyroid diagnostic criteria and the corresponding results. (**a**) Scheme depicting the workflow: first, radiologists extract features from the ultrasound images and a convolutional neural network of arbitrary architectural design produces primary diagnosis results as a separate feature to construct a candidate feature set and then use a feature selection module that optimizes feature weights to generate the thyroid diagnostic criteria and finally return the diagnosis. The risk stratification module ensures that the malignancy rate for each risk category is within the corresponding specified range. (**b**) The neural network architecture of our proposed GAFM model consists of an input layer, embedding layer and interaction layer identical to an AFM, with an additional gate mechanism to filter out minimally relevant second-order interacting features. (**c**) Bar plot of the mean AUC values together with the corresponding standard deviations from 10-fold feature-stratified cross-validation experiments for diagnosing thyroid nodules using different methods. (**d**) The correlation between the assigned score of each selected feature by the GAFM model and the corresponding odds ratio. (**e**) The probability density functions of HAIbrid-TIRADS scores computed based on GAFM model for benign and malignant samples. (**f**) The distributions of samples according to our risk classifications. (**g**) Table of score ranges for risk classifications.

This part mainly describes the construction of candidate feature set from features extracted by radiologists and diagnosis results of a convolutional neural network for establishment of interpretable thyroid diagnosis criteria and malignancy risk stratification, as illustrated in Figure 1a.

The features extracted by radiologists are descriptions of ultrasound images as text data using feature dictionary defined in TIRADS. Each sample is represented by structured data in consistent order. One-Hot Encoding is used to convert text data to feature vectors, using N-bit status registers to encode N states, with each state having an independent register bit, and only one bit being valid at any time. For absent feature description, 0 was used to encode the feature to ensure consistent vector dimension.

In our study, the collected samples were labeled on the basis of Kwak-TIRADS and our defined extra features, most of which were already described in the published C-TIRADS [7] except features defined in color Doppler images, to explore possibilities to further improve the diagnosis. The complete candidate features are listed in Appendix A Table A1.

The diagnosis results from CNN-based CADx were included as an additional candidate feature for constructing candidate feature set together with human-extracted features. This is different from the conventional procedure where image features are extracted via a CNN model for later feature fusion and classifications.

### 2.3. Feature Selection Based on AFM and GAFM Model

The candidate features were thereafter used to construct weighted second-order feature interactions using either AFM or our proposed variant GAFM module according to the individual contribution of each feature to the diagnosis. The difference between AFM and GAFM is that an additional gate mechanism is introduced to only keep the top-ranked features according to their weights. The inclusion of the gate mechanism is because some insignificant second-order features, if not removed, can interfere the learned weights of other features and return sub-optimal combinations of features for the ultimate thyroid malignancy classifications. The architecture of our proposed GAFM model is illustrated in Figure 1b. The input of the model is the feature vector from the candidate feature set and we denote the feature vector as ***X***.

#### 2.3.1. Embedding Layer

This layer adopts a sparse representation for input features and embeds each non-zero feature into a dense vector, which can be represented as

(1) $$e_{i}=V_{i}x_{i},i=1,2,...,n,$$

where $x_{i}$ is a non-zero feature in the feature vector ***X*** and *n* is the number of the non-zero features. The output of the embedding layer is then the concatenation of multiple embedding vectors as:

(2) $$E=\left[e_{1},e_{2},...,e_{n}\right],i=1,2,...,n$$

#### 2.3.2. Interaction Layer

For pairwise interactions of *n* vectors, there are *n*(*n*-1)/2 interacting vectors, each of which is the element-wise product of two distinct vectors representing their interaction. This can be expressed as:

(3) $$\left[\langle e_{1},e_{2}\rangle ,\langle e_{1},e_{3}\rangle ,...,\langle e_{n-1},e_{n}\rangle \right]$$

where $e_{i}\;$ is the feature embedding and <□,□> represents the inner product of two vectors.

#### 2.3.3. Attention Layer

The attention mechanism of the GAFM model is the same as AFM, which parameterizes the attention score with a multi-layer perceptron (MLP). The input of the gate-attention network is the pairwise interacting vectors, while the output is the attention scores, a vector whose dimension is consistent with the number of interacting vectors. The attention score of each feature interaction in the embedding space $a_{\left(i,j\right)}$ for the entire sample on average $a_{\left(i,j\right)}$ representing the feature importance is given by:

(4) $$\alpha_{\left(i,j\right)}=\frac{1}{N}\sum_{N}a_{\left(i,j\right)}$$

The gate mechanism introduced in GAFM model sets a gate operation for each second-order interacting feature, and the number of gate operations are consistent with that of the interacting features. When the weight of the interacting feature is greater than the preset threshold, the gate state is open, and second-order interacting feature is retained, otherwise the opposite.

### 2.4. Model Training

We propose a two-step training procedure for our GAFM model. The first step is similar to the training process of an AFM model and there is no gate mechanism involved. After the model converges, we use the gate mechanism to remove minimally relevant second-order interacting features. The second step is to re-train the model after the gate operation with the remaining second-order interacting features and all first-order ones. The output of gate-attention layer can be expressed as:

(5) $$L_{GAFM}=<W,X>+\sum_{(i,j)\in \Gamma }a_{\left(i,j\right)}<e_{i},e_{j}>$$

where $\Gamma =\{(i,j)|\alpha_{\left(i,j\right)}>\varepsilon ,i,j=1,2,...,n\}$ is the subscript set of the remaining second-order interacting features after gate operation with a threshold of ε. For conventional AFM, no gate operation is applied, therefore all second-order interacting features are kept. The output of the GAFM model can be expressed as:

(6) $$y_{GAFM}=sigmoid\left(L_{GAFM}\right)$$

The objective function is the cross-entropy loss function expressed as:

(7) $$L(y,y_{GAFM})=-y\log(y_{GAFM})-(1-y)\log(1-y_{GAFM})$$

After the model converges, we normalize the weights of all remaining features through the softmax function, then rank the coefficient of each feature in the model and select the top-ranked features. Following the design principle of Kwak-TIRADS, 15 top-ranked features were selected. The expression of the softmax function is as follows:

(8) $$f{\left(X\right)}_{j}=\frac{e^{x_{j}}}{\sum_{k=1}^{K}e^{x_{k}}}$$

where X is a K-dimensional vector, $x_{j}$ is the *j-th* element and $f{\left(X\right)}_{j}$ is the softmax function value of feature $x_{j}$ .

### 2.5. Risk Stratification

Once the feature selection and corresponding weight for each selected feature is determined after the model training, a 1–5 risk stratification system (including 4A, 4B and 4C) is constructed. This is performed by summing the weights for each sample *j*:

(9) $${Score}_{j}=\sum_{i\in O}W_{i},$$

in which *O* represents the feature set for sample *j* that includes all assigned features by radiologists and the diagnosis by the CNN-based CADx system, while $w_{i}$ represents the score for each feature *i* of sample *j*.

The malignancy rate for each summed score S ( $MR_{Score}$ ) is computed as follows:

(10) $${MR}_{Score}=\frac{M_{Score}}{M_{Score}+B_{Score}},$$

where $M_{Score}$ represents the number of malignant samples with a summed score S while $B_{Score}$ represents the number of benign samples with a summed score S. Thereafter, the score range $\left[a_{i},a_{i+1}\right)$ for each assigned risk category *i* according to the malignancy rate illustrated in the risk stratification module in Figure 1 is determined. Remember that during model training, all weights are normalized with their sum equals to 1. However, this is not user-friendly to radiologists. To mimic a conventional TIRADS score system, a multiplication factor to weight is manually optimized and each obtained weight is then rounded to an integer. 

### 2.6. Control Experiments

To evaluate the effectiveness of our proposed diagnostic framework that takes advantages of both CNN-based CADx and radiologist-extracted features for thyroid nodules, we designed a series of control experiments as summarized in Table 1.

Our proposed diagnostic framework to establish new understandable TIRADS criteria for thyroid nodules relies on using radiologist-extracted features according to TIRADS criteria and the diagnosis results from CNN-based CADx. As a simple ablation study to verify the significance of our HAIbrid approach, we included the diagnosis by radiologists according to Kwak-TIRADS criteria (method 1) or with a subsequent LR classifier (method 2), or with additionally defined candidate features (method 3), the diagnosis by conventional CADx (method 4), a simple integral approach that combine the diagnoses by radiologists and a CADx system by returning always the higher malignancy (method 5) or a plain integral approach that combines the features defined in TIRADS and the diagnosis results from CADx followed by using LR merely for classification (method 6) or as both a feature selector and classifier (method 7). To elucidate the significance of second-order feature interactions and the importance of further feature weight optimization, we included conventional AFM (method 8), our proposed GAFM variant (method 9) and a conventional method ignoring second-order feature interactions (method 7).

For verifying the results obtained from the cross-validation experiments, a CNN-based CADx system, AI-SONIC^TM^ Thyroid of software version 4.0 (2020) developed by Demetics Medical Technology (Hangzhou, China), was tested to check whether the proposed integration framework could also bring benefits to an independently trained CNN model.

### 2.7. Statistical Analysis

For cross-validation experiments, we used pairwise two-tailed t-test to evaluate whether the differences in mean AUC values between the GAFM model and control experiments were statistically significant. For comparison of two CNN models integrated through the GAFM-HAIbrid strategy, we subdivided the ultrasound images of 500 nodules to two groups, each of which were further partitioned to five subsets for both intra-group and cross-group statistical comparisons using the feature stratified partition method mentioned above. For intra-group comparisons of different diagnostic methods, as the dataset partitions were kept identical, pairwise two-tail t-tests were used for statistical evaluations. For cross-group comparisons, standard two-tail t-tests were used instead. 

## 3. Results

We first performed a feature-stratified 10-fold cross-validation experiment on retrospectively collected 3002 nodules (1270 malignant ones) from our nationwide database contributed by 131 member hospitals. From the results, it can be seen that combining the CADx with TIRADS, denoted as HAIbrid+LR_0_ (where LR was used merely as a classifier without feature exclusion) outperformed each separate method, i.e., Kwak-TIRADS and CADx (Supplementary Figure S1). Interestingly, the inclusion of extra primary candidate features made no difference when using LR as a classifier. In contrast, enabling feature selection capability of LR while providing extra candidate features with HAIbrid^+^-LR_1_ improved the diagnosis compared to the case of HAIbrid-LR_0_ (0.893 ± 0.0017 vs 0.881± 0.0025). Furthermore, AFM-based methods including our proposed variant GAFM achieved significantly better performances (Figure 1c and Supplementary Figure S1) than the HAIbrid^+^-LR_1_. In addition, incorporating a gate mechanism to AFM by removing minimally relevant second-order features using GAFM significantly improved the diagnosis (*p*-value = 1.4 × 10^−5^ in Supplementary Figure S1).

The resulting TIRADS criteria, referred as HAIbrid-TIRADS which takes diagnosis from CNN-based CADx as an additional feature reweighted together with ultrasonographical features noted by radiologists, are listed in Supplementary Table S1 with the corresponding scores returned by the GAFM model for primary features and second-order feature interactions. The distribution of the summed scores and that of the corresponding risk stratifications for the benign and malignant samples confirmed by pathological examinations are shown in Figure 1e and Figure 1f, respectively. The optimal threshold chosen by maximizing F1 score for thyroid nodule diagnosis based on HAIbrid-TIRADS scores was found to be 9 (for which 10 and above were considered suspicious to be malignant, Supplementary Figure S2); at this threshold, the precision and sensitivity were found to be 0.921 and 0.912 with the false positive and false negative rate being 0.102 and 0.088, respectively.

It is worth-noting that our GAFM model identified the second-order feature interaction concerning vasculature localization and morphology (noted by radiologists from Doppler ultrasound images in Supplementary Figure S3), i.e., being mainly perinodularly distributed and untwisted (OR = 2.349) to be important for thyroid nodule classifications. However, these vasculature-related features defined by radiologists in this study played no significant roles when fed directly to conventional classification methods such as LR, in which second-order feature interactions were ignored (Figure 1c and Supplementary Figure S1). On the ultrasound image test set of 500 extra thyroid nodule, the OR of the vasculature-related second-order feature interaction was 2.41, consistent to the OR calculated from the retrospectively collected 10-fold cross validation set.

Based on the 10-fold cross-validation experiments, another identified candidate feature in this study was punctate echogenic foci of undetermined significance, defined according to C-TIRADS [7], was found to have an OR of 1.891 (Supplementary Table S1). However, on the test set of 500 extra thyroid nodules, the OR was 1.27 instead.

**Figure 2:**
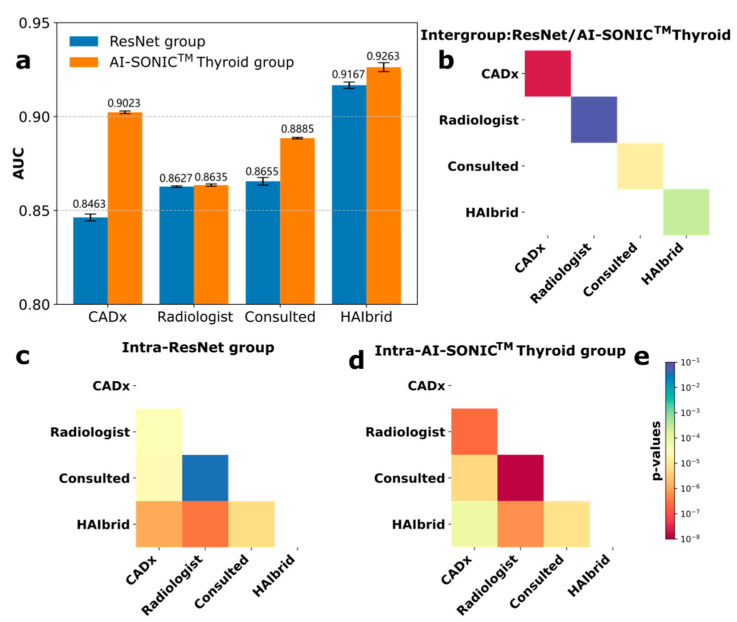
Comparison of two feature-stratified groups of equal sizes (250 cases each) to evaluate the importance of applying GAFM-based HAIbrid approach for improving thyroid nodule diagnosis performances of two CADx systems, the trained ResNet101 and commercial AI-SONIC^TM^ Thyroid. (**a**) The barplots of the diagnosis AUCs for CADx, radiologists, radiologists consulting the respective CADx and the HAIbrid approach in each group. (**b**) *p*-value heatmap for inter-group comparison. (**c**) *p*-value heatmap for the intra-group comparison for the ResNet group as well as for the AI-SONIC^TM^ Thyroid group (**d**). (**e**) The common *p*-value color-bar in logarithmic scale shared by (**b**–**d**).

Furthermore, we applied the GAFM-HAIbrid method on two different CNN models to assess the effectiveness of this approach on the test cohort by dividing the ultrasound images of 500 thyroid nodules to two groups of equal sizes in a feature-stratified manner, one for the trained ResNet101 model and the other for a CNN-based CADx system, AI-SONIC^TM^ Thyroid. It is clear that in both groups the HAIbrid results exceeded the diagnoses by the radiologists alone by large margins (Figure 2a) and were significantly better than the standalone CADx models (Figure 2a,c,d). The performances of the radiologists in these two groups were comparable (*p*-value = 0.065, Figure 2b). Interestingly, by combining radiologists’ diagnoses through our HAIbrid approach, the performance gap between the under-performing ResNet and the AI-SONIC^TM^ Thyroid system (Figure 2a) was largely closed-off (from 0.8463 ± 0.0020 versus 0.9023 ± 0.0008 to 0.9167 ± 0.0019 versus 0.9263 ± 0.0027) despite a still measurable statistical difference (*p*-value ~ 2 × 10^−4^).

## 4. Discussion

From the 10-fold cross validation experiments on retrospectively collected thyroid nodule ultrasound images, feature selection and weight reassignment in general can make an influential impact on the performance of the resulted computer-aided TIRADS criteria. For instance, enabling feature selection capability of conventional LR while providing extra candidate features improved the diagnosis compared to the case where LR was only used as a classifier, suggesting the relevance of feature selections on additional features for improving thyroid nodule diagnosis. Furthermore, the score of each selected feature correlated very well with their corresponding odds ratios (ORs), supporting that the learned scores reflect the relative malignancy risks of each feature. Implementing AFM-based feature selection modules especially our proposed GAFM variant within the HAIbrid framework that consider second-order interacting features further improved the diagnostic performances.

The GAFM-HAIbrid model identified a second-order vascular-related feature being mainly perinodularly distributed and untwisted. At first glance, it may appear contradictory to the notion that the thyroid nodule malignancy was more associated with the intranodular vascularity given by a study of 402 nodules, in which 23 were malignant [8]. However, according to a recent quantitative study of regional vascularity of 111 thyroid nodules, in which 27 were malignant, the mean vascularity index of peripheral region of the malignant nodules significantly higher than that of the benign nodules. In addition, the authors that suggested the contrary had only the binary definition of intra- and peri-nodular vascularity, different from our definition of five cases, and their identified association between regional vascularity and nodule malignancy may well be sample-dependent. In our study, 3002 nodules (including 1270 malignant ones) were continuously collected from 131 member hospitals of the CAAU, and should contain less sampling bias. Furthermore, though there was a claim that markedly chaotic central and peripheral vasculature was considered more suspicious for malignancy [28], to our humble knowledge, there has not been a quantitative study about what vasculature localization patterns and morphology concurrently correlate with thyroid nodule malignancy. Vasculature-related second-order feature from this test set was found to be 2.41 (not much different from 2.349), confirming the validity of it as a useful malignancy marker.

Based on the 10-fold cross-validation experiments, a first-order feature “punctate echogenic foci of undetermined significance” was also identified by the GAFM-HAIbrid method with an OR of 1.891. Its OR was different from the OR of 0.944 in the original C-TIRADS paper, where it was considered insignificant [7]. This is most likely due to differences in samples distribution though substantial overlapping cases could be expected as the data used in this study came from the same database maintained by CAAU. In the test cohort of the 500 extra nodules, its OR was 1.27 instead, suggesting the contribution of this feature for malignancy prediction was unstable. One explanatory factor to the observed high degree of variability is that all sample data were periodically uploaded to the CAAU database while different contributing hospitals could have different operational cycles. Nevertheless, this complicating factor had a negligible effect on our identified vasculature-related second-order feature.

It is also interesting to note that our test dataset results show that combining radiologists’ diagnoses through our HAIbrid approach had a greater impact on the under-performing ResNet such that the standalone performance gap compared to AI-SONIC^TM^ Thyroid system was largely closed-off. This suggests that standardizing the incorporation of radiologists’ expertise to CNN-based CADx systems may outweigh the improvement of standalone CADx systems. In contrast, by inspecting the developments of high-profile deep learning algorithms from a historical perspective, it can be seen that they typically outperformed the previous state of the art algorithms by 1–3% [25,29,30,31,32]. It should also be noted that the better performing standalone EfficientNet-based AI-SONIC^TM^ Thyroid system than the ResNet101 model may have benefited more from much larger available training samples than we had in this study. Nevertheless, this indicates that from a practical perspective, pushing the performance of a standalone CADx system to exceed senior radiologists’ diagnostic performance for the real-world clinical workflow may no longer be an urgent need.

At the same time, the results show that our HAIbrid approach did not require the CNN-based CADx to be superior to radiologists for diagnosis performance improvement. This is different from other publications where the improvement of radiologists’ diagnosis by consulting the results from CADx systems demands these systems to be more accurate than the radiologists [10,33]. Our approach instead can be perceived as an ensemble learning model [10,34] that is based on two constituent weaker learners, although one of them is radiologists. This methodology puts a softer requirement on the constituent raters that the predictions by CNN-based CADx systems are independent of the radiologists. Or in other words, there exists valuable complementary information that can be utilized to improve the final diagnosis. This is easier to fulfill because these systems are usually trained according to results from pathological examinations rather than radiologists’ ratings in reference to a set of TIRADS criteria [35].

It is however desirable to relieve the burden of radiologists as much as possible. It is preferable to obtain the TIRADS features from another CNN system as done in reference [35] and to allow human intervention if needed. With this minimally human-intervened variant of HAIbrid approach, favorable image feature interpretability, diagnosis standardizability, and user-friendliness are simultaneously achieved. This is certainly of interest, but requires a large dataset with tremendous annotation efforts to train a CNN system to achieve accurate multi-class classification results. Other limitations of this work include that we have not tested the improvement of diagnostic accuracy for junior radiologists but focused on senior radiologists. Our consideration for this omission is mainly due to the fact that we focus on identifying novel malignancy-relevant features. In addition, in the clinical routine, junior radiologists’ diagnoses need to be double-checked by a senior radiologist before vital decisions are made. We also have good reasons to believe that our framework will improve their diagnoses significantly also. It has been shown in numerous studies that improving senior radiologists’ diagnostic accuracy is more difficult than junior radiologists by using a CNN system [10,34]. For single features, the highest weight was assigned to the prediction by the CNN system in our GAFM-HAIbrid model (Supplementary Table S1). As the two CNN systems evaluated in this work have equivalent diagnostic performances to senior radiologists or even better, it is expected that having a high weight on the CADx system when integrating with the junior radiologists’ diagnoses will certainly be helpful as well for them. A third perceivable limitation is that an international multicenter study would be preferred to validate whether our findings in this work are universally applicable. However, this is for future investigation.

## 5. Conclusions

To conclude, our HAIbrid approach allows integration of CNNs of arbitrary architectural designs, combines thyroid nodule diagnosis results from CNN-based CADx system and radiologist-defined features, permits the establishment of novel human interpretable TIRADS criteria that outperform the original diagnostic methods and requires no extra efforts from radiologists to adapt and integrate to their clinical workflow. Our proposed GAFM feature selection method enables the identification of clinically relevant second-order feature interactions, which have been overlooked by radiologists and conventional feature selection methods. We anticipate that our integrated approach can be generally applicable for medical imaging-based diagnosis of other diseases beyond ultrasonography as long as radiologists may consult the second opinion from a CNN-based CADx system and our proposed GAFM model can be presumably adopted to identify potentially important second-order feature interactions that are overlooked in existing diagnostic criteria.

## 6. Patents

A patent application has been filed with publication number CN113889229A, and publication date 24 January 2022.

## Figures and Tables

**Table 1:** Experiment ID, the corresponding method and short descriptions.

Exp. #	Method	Description
1	Kwak-TIRADS	Kwak-TIRADS criteria were followed by radiologists to extract features and to evaluate thyroid nodules.
2	Kwak-TIRADS + LR_0_	Radiologists followed Kwak or candidate TIRADS^+^ to extract features; LR worked as a classifier, denoted as LR_0_.
3	TIRADS^+^-LR_0_	
4	CADx (ResNet101)	ResNet101 was used as an exemplar model for CADx.
5	Kwak-TIRADS/CADx	Return diagnosis of higher malignancy from either method.
6	HAIbrid-LR_0_	Hybrid denotes combining features from TIRADS and diagnosis results from CNN-based CADx for classifications using LR merely as a classifier, denoted as LR_0_.
7	HAIbrid^+^-LR_1_	Hybrid+ denotes the combination of features defined in TIRADS^+^ with diagnosis results from CADx; LR_1_ *, AFM or GAFM when each corresponding method was used as the feature selector and classifier simultaneously.
8	HAIbrid^+^-AFM	
9	HAIbrid^+^-GAFM	

* When using LR_1_ as both a feature selector and classifier, the cross-entropy loss function is used as the cost function to learn the weights of each feature vector through stochastic gradient descent. After the model converges, the weight of each feature is ranked and the top-ranked features selected for later thyroid diagnosis.

## Data Availability

The data that support the findings of this study are available on reasonable request from corresponding author J.Z. after formal approval by the concerned Chinese regulating authorities.

## Supplementary Materials

The following supporting information can be downloaded at: https://www.mdpi.com/article/10.3390/cancers14184440/s1. Figure S1. Bar plot of the mean AUC values in diagnosing thyroid nodules from 10-fold cross-validation experiments on 3002 nodules and the statistical comparisons; Table S1. Our HAIbrid-TIRADS criteria returned by the proposed GAFM model for thyroid nodule malignancy risk stratification from the cross-validation cohort; Figure S2. The identification of the optimal threshold to separate malignant from benign samples based on F1 score computed using our HAIbrid-GAFM model; Figure S3. Representative images for additionally defined vasculature-related features.

## Appendix A. Candidate Ultrasonographical Features

**Table A1:** Our defined candidate features (TIRADS+) of thyroid nodules in ultrasound images to establish a clinically relevant HAIbrid-TIRADS. The features shown in light gray are extra features defined with the aim to identify new potentially useful features for thyroid diagnosis.

Feature	Definition	Feature	Definition
***Orientation (Shape)***	Parallel (Wider-than-tall)	***Homogeneity of echo intensity***	Homogeneous
	Vertical (Taller-than-wide)		Inhomogeneous
***Margin***	Circumscribed	***Vasculature*** ***localization***	Avascular
	Ill-defined		Perinodular
	Irregular		Mixed
	Extra-thyroidal extension/micro-lobulated		Mainly intranodular
***Composition***	Solid		Mainly perinodular
	Predominantly solid	***Vascular*** ***morphology***	Not twisted
	Mixed cystic and solid		Twisted
***Macro-calcification***	Present	***Halo thickness***	Thin
	Absent		Thick
***Echogenicity***	Hyperechoic	***Posterior echo features***	Absent
	Isoechoic		Enhanced
	Hypoechoic		Shadowing
	Marked hypoechoic		Mixed
***Echogenic foci***	Micro-calcifications	Note: (1) Micro-calcifications correspond to punctate echogenic foci with or without shadowing; (2) Punctate echogenic foci of undetermined significance correspond to punctate echogenic foci without shadowing or comet-tail artifacts, therefore it is difficult to tell whether it is micro-calcification or colloid [7].	
	Comet-tail artifacts		
	Peripheral calcifications		
	No punctate echogenic foci		
	Punctate echogenic foci of undetermined significance [7]		
