# Supplementary material for: Novel Human Artificial Intelligence Hybrid Framework Pinpoints Thyroid Nodule Malignancy and Identifies Overlooked Second-Order Ultrasonographic Features
Source: Cancers (Basel). 2022 Sep 13;14(18):4440. doi: 10.3390/cancers14184440 (PMC9497166; doi:10.3390/cancers14184440)
Supplement: Supplementary file 1 [file cancers-14-04440-s001.zip › cancers-1841959-supplementary.pdf]

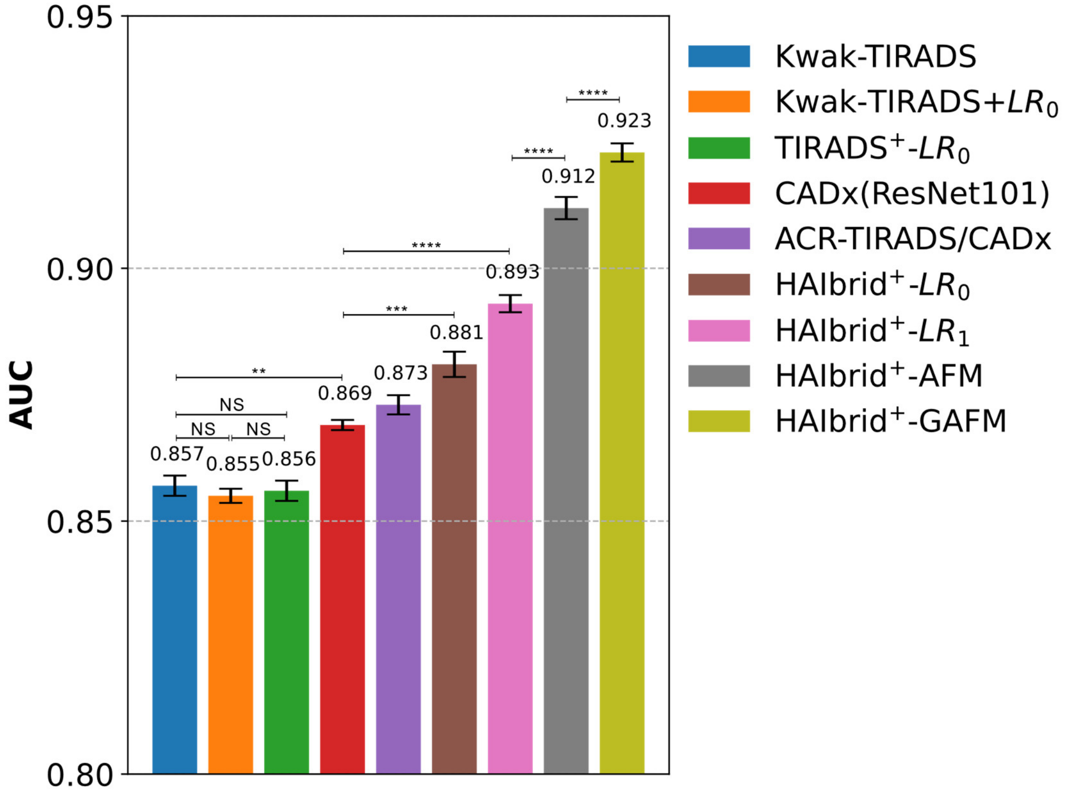

Figure S1. Bar plot of the mean AUC values in diagnosing thyroid nodules from 10-fold cross-validation experiments on 3002 nodules and the statistical comparisons. The mean AUC values are provided at the top of each bar and the corresponding standard deviations are displayed as error bars. For the sake of clarity, only a few statistical comparisons with the associated significance levels (indicated by the number of stars) are provided and "NS" sign indicates non-significant difference between compared cases. The meanings of LR_0, LR_1 and the + sign are referred to descriptions in Appendix Table 1.

Table S1. Our HAIbrid-TIRADS criteria returned by the proposed GAFM model for thyroid nodule malignancy risk stratification from the cross-validation cohort.

Feature		Score	Odds ratio
Malignant as diagnosed by CADx		8	10.923
Shape	Taller than wide	3	4.206
	Wider than tall	0	1.045
Margin	Extra-thyroidal extension	4	6.102
	Irregular	3	5.686
	Ill-defined	1	2.107
Composition	Solid	3	3.357
	Predominantly solid	1	2.312
Echogenicity	Marked hypoechoic	4	7.812
	Hypoechoic	1	2.713
	Isoechoic	0	1.054
	Anechoic	0	1.023
Echogenic foci	Micro-calcifications	4	6.834
	Punctate echogenic foci of undetermined significance	1	1.891
vasculature	Mainly perinodular and not twisted	2	2.349

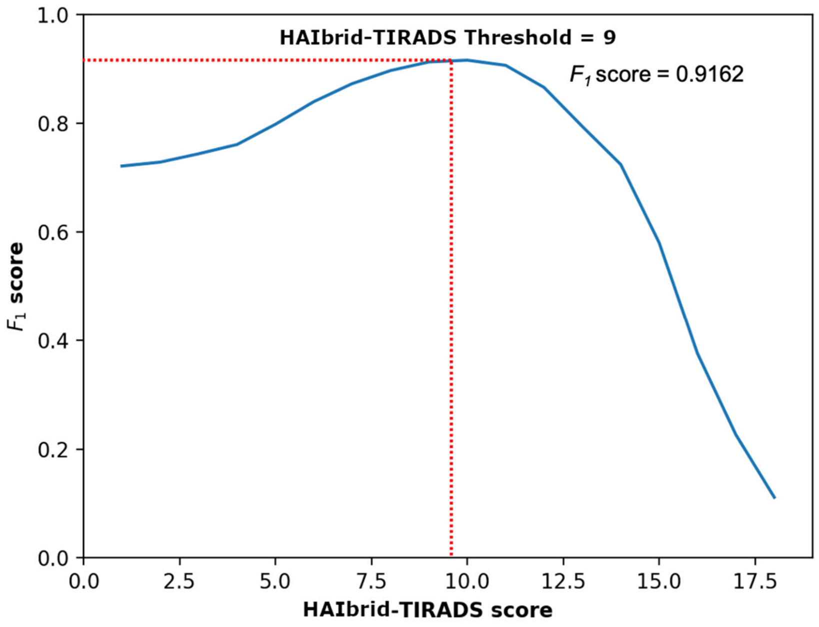

Figure S2. The identification of the optimal threshold to separate malignant from benign samples based on F1 score computed using our HAIbrid-GAFM model.

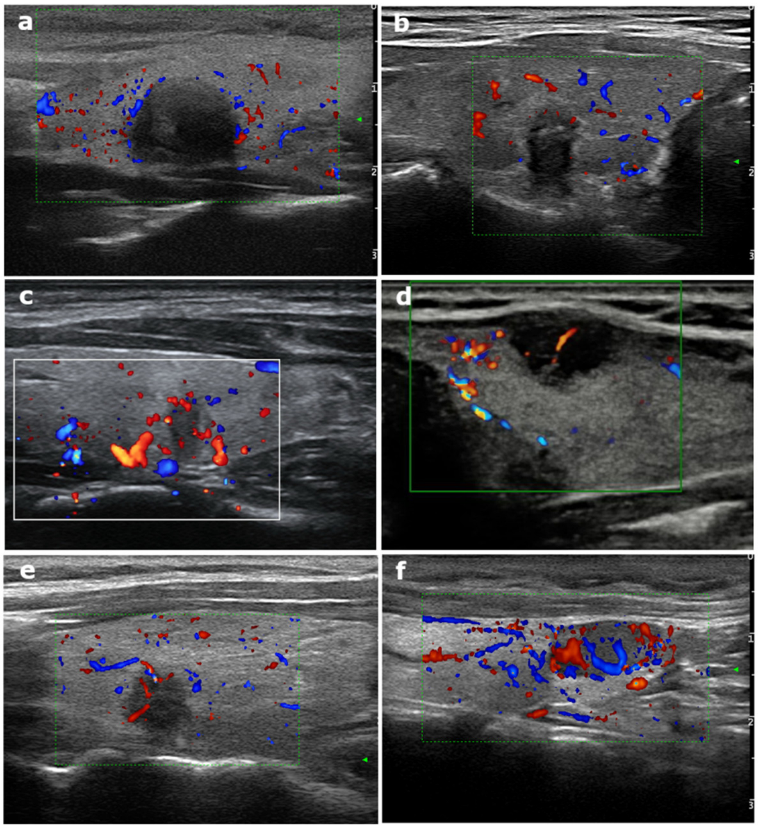

Figure S3. Representative images for additionally defined vasculature-related features. (a) Circumscribed solid nodule with avascularity diagnosed as nodular goiter (b) Irregular solid nodule with untwisted perinodular vascularity diagnosed as papillary thyroid carcinoma (c) Irregular solid nodule with twisted mainly perinodular vascularity diagnosed as papillary thyroid carcinoma (d) Irregular solid nodule with untwisted mainly intranodular vascularity, diagnosed as papillary thyroid carcinoma (e) Irregular solid nodule with untwisted mainly perinodular vascularity, diagnosed as papillary thyroid carcinoma (f) Circumscribed solid nodule with twisted mixed vascularity, diagnosed as nodular goiter.
